# Supplementary material for: Structure of recombinant formate dehydrogenase from Methylobacterium extorquens (MeFDH1)
Source: Sci Rep. 2024 Feb 15;14:3819. doi: 10.1038/s41598-024-54205-7 (PMC10869683; doi:10.1038/s41598-024-54205-7)
Supplement: Supplementary file 1 — Supplementary Information. [file 41598_2024_54205_MOESM1_ESM.pdf]

SUPPLEMENTARY INFORMATION

Structure of recombinant formate dehydrogenase from *Methylobacterium extorquens* (MeFDH1)

Junsun Park[1†], Yoonyoung Heo[2†], Byoung Wook Jeon[3†], Mingyu Jung[1], Yong Hwan Kim[3*], Hyung Ho Lee[2*], Soung-Hun Roh[1*]

[1]Department of Biological Sciences, Institute of Molecular Biology and Genetics, Seoul National University, Seoul 08826, Republic of Korea

[2]Department of Chemistry, College of Natural Sciences, Seoul National University, Seoul 08826, Republic of Korea

[3]School of Energy and Chemical Engineering, Ulsan National Institute of Science and Technology, Ulsan, 44919, Republic of Korea

[*]Correspondence to:hyungholee@snu.ac.kr; (H.H.L.); metalkim@unist.ac.kr (Y.H.K.); shroh@snu.ac.kr (S.-H.R.)

[†]These authors contributed equally to this work.

LIST OF SUPPLEMENTAL INFORMATION

Supplementary Table S1. Cryo-EM data collection, refinement and validation statistics

Supplementary Figure S1. Cryo-EM data processing and workflow

Supplementary Figure S2. Structural comparison of the subdomains in MeFDH1

Supplementary Figure S3. Structural comparison of the subdomains in MeFDH1

Supplementary Figure S4. The comparison between wild-type versus recombinant MeFDH1

Supplementary Figure S5. The cap domain and NADH binding site of MeFDH1

Table S1. Cryo-EM data collection, refinement and validation statistics

	MeFDH1 (holoenzyme)<br>(EMDB-30995)<br>(PDB 7E5Z)
**Data collection and processing**	
Magnification	92,000
Voltage (kV)	200
Electron exposure (e–/Å^2)	50
Defocus range (μm)	1.2-2.0
Pixel size (Å)	1.08
Symmetry imposed	C1
Initial particle images (no.)	2,188,878
Final particle images (no.)	453,262
Map resolution (Å)	2.83
FSC threshold	0.143
Map resolution range (Å)	2.4-3.2
**Refinement**	
Initial model used (PDB code)	6TGA
Model resolution (Å)	3.0
FSC threshold	0.5
Model resolution range (Å)	2.4-3.2
Map sharpening *B* factor (Å^2)	-144
Model composition	
Non-hydrogen atoms	10,393
Protein residues	1,331
Ligands	10
B factors (Å^2) [min/max/mean]	
Protein	4.41/74.52/20.96
Ligand	5.65/87.43/21.63
R.m.s. deviations	
Bond lengths (Å)	0.026
Bond angles (°)	0.579
Validation	
MolProbity score	2.00
Clashscore	14.03
Poor rotamers (%)	0.00
Ramachandran plot	
Favored (%)	95.03
Allowed (%)	4.97
Disallowed (%)	0.00

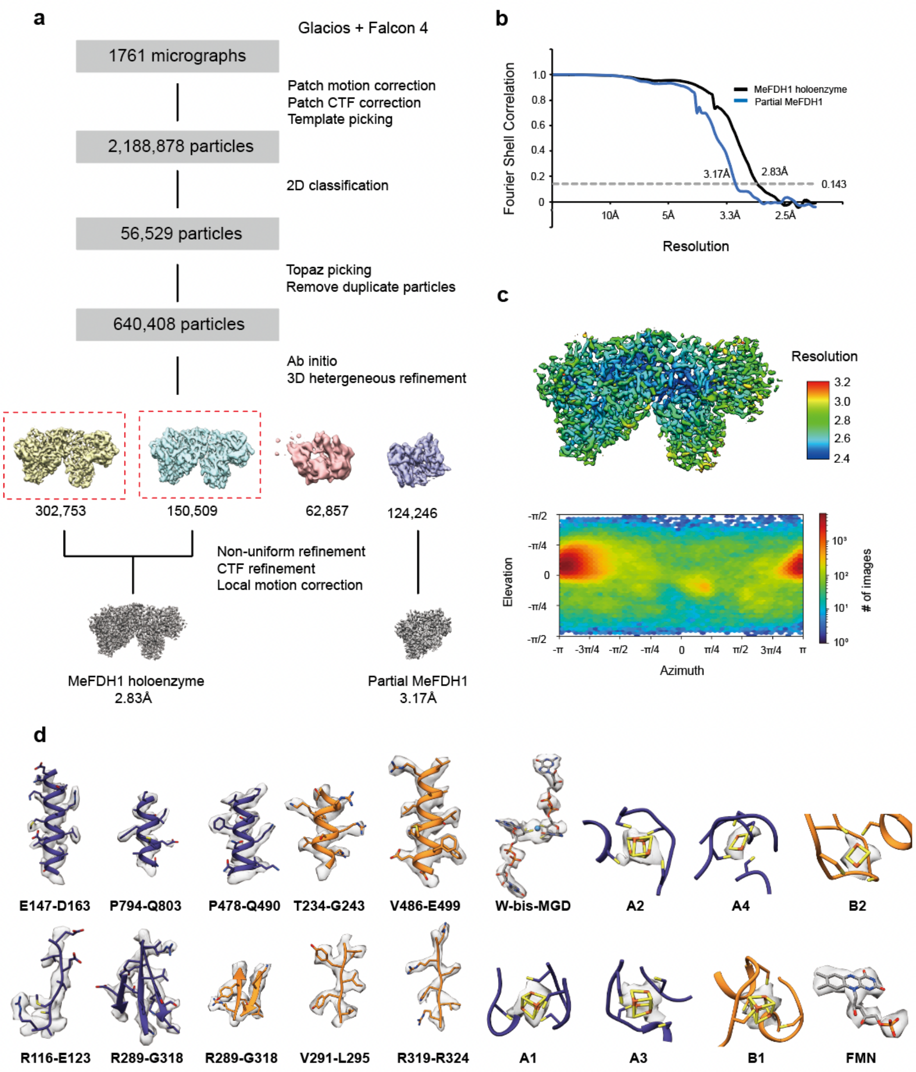

Supplementary Figure S1. Cryo-EM data processing and workflow

(**a-b**) Data processing workflow and Fourier shell correlation (FSC) curve plot. (**c**) Local resolution map and Euler angle distribution. (**d**) Cryo-EM density and model fitting.

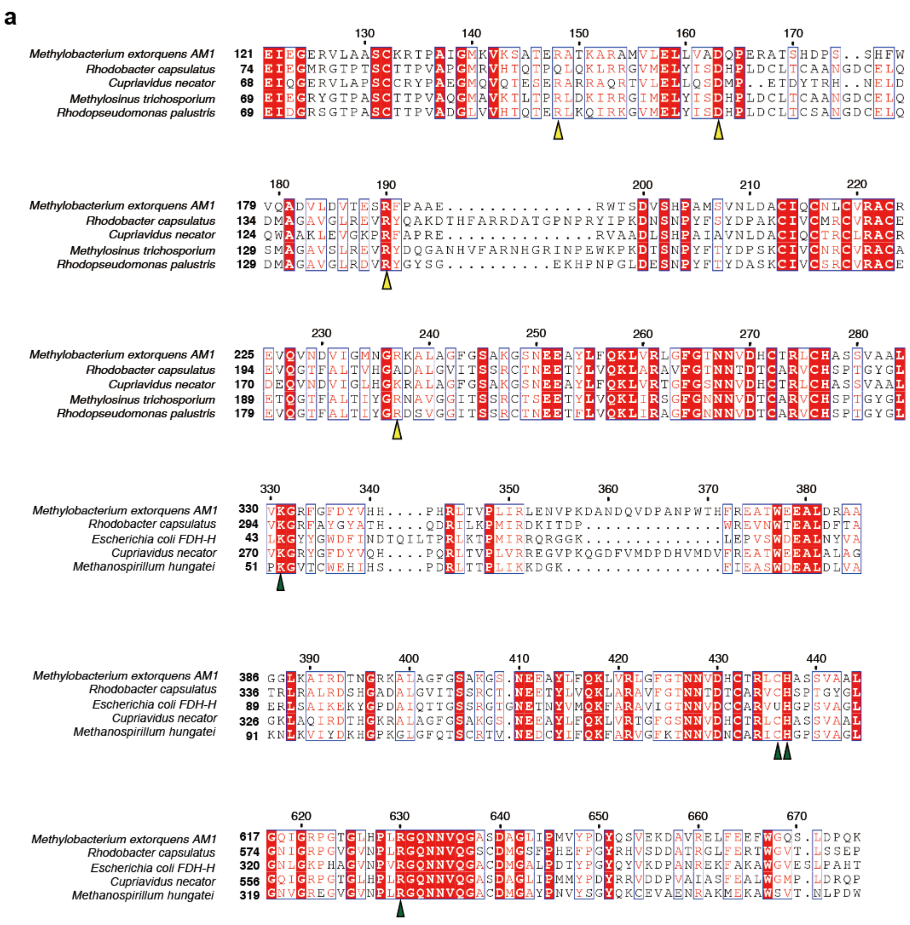

Supplementary Figure S2. Structural comparison of the subdomains in MeFDH1

(**a**) Clustal W[38] sequence alignments of the alpha subunits of formate dehydrogenase from *M. extorquens* AM1 (UniProtKB/Swiss-Prot accession number S31C5ATT7), *Cupriavidus necator* (UniProtKB/Swiss-Prot accession number Q0K0I9), *Escherichia coli* (UniProtKB/ Swiss-Prot accession number P07658), *Rhodobacter capsulatus* (UniProtKB/Swiss-Prot accession number D5AQH0) and *Methylosinus trichosporium* (UniProtKB/Swiss-Prot accession number 0A2D2D2N4). Identical residues are highlighted in red with white letters, and functionally conserved residues (80%) are highlighted in white with red letters. Residues forming salt bridges at the interface of alpha and beta subunits are indicated by yellow arrows below the sequences. Residues near active sites (W-bis-MGD binding site) are indicated by green arrows below the sequences.

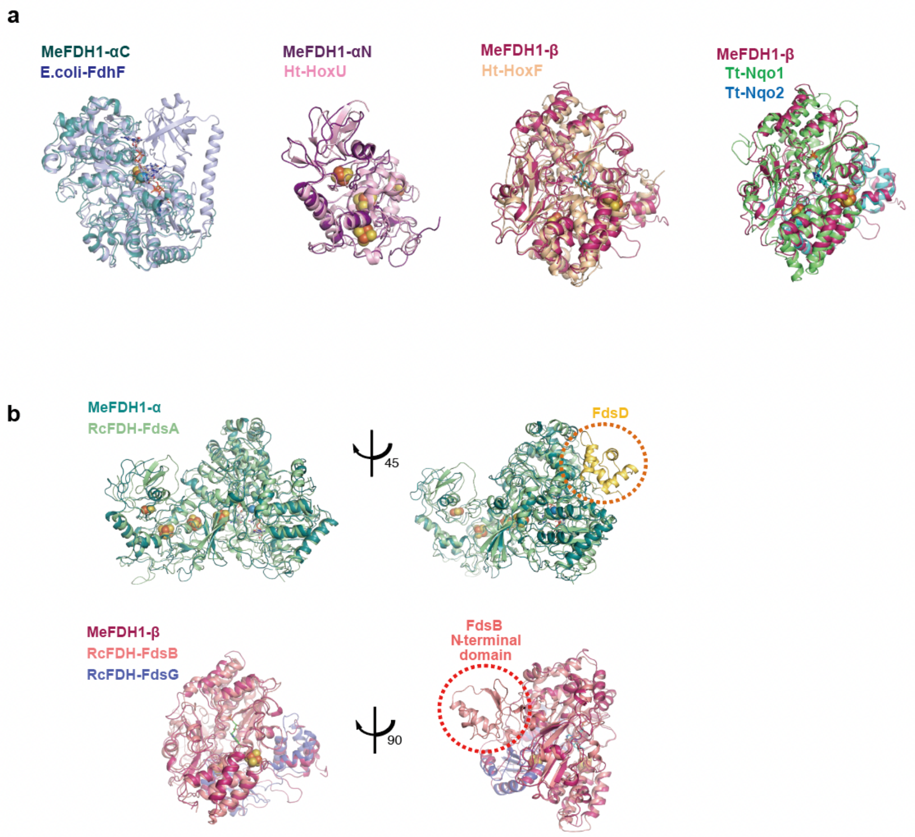

Supplementary Figure S3. Structural comparison of the subdomains in MeFDH1

(**a**) Structural comparisons of MeFDH1-αC (residue 289-999) vs. E.coli-FdhF, MeFDH1-αN (residue 1-289) vs. Ht-HoxU (PDB ID: 5XF9), MeFDH1-β vs. Ht-HoxF (PDB ID: 5XF9), and MeFDH1-β vs. Tt-Nqo1 vs. Tt-Nqo2 (PDB ID: 2FUG). (**b**) Structural comparison of MeFDH1-α (blue-green) vs. the FdsA subunit of RcFDH (light green). Structural comparison of MeFDH1-β (magenta) vs. the FdsB-FdsG complex from RcFDH (PDB ID:6TGA) (salmon and light blue, respectively)

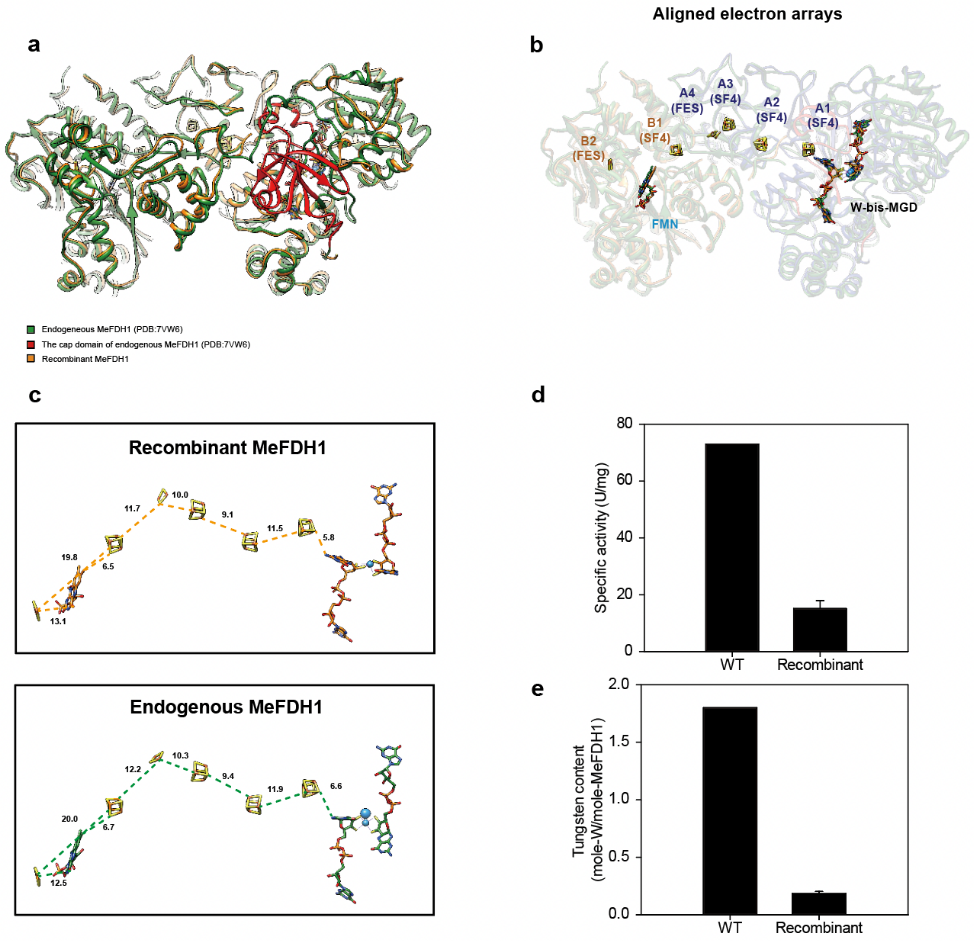

Supplementary Figure S4. The comparison between wild-type versus recombinant MeFDH1.

(**a**) The aligned atomic models of recombinant versus endogenous MeFDH1. The red domain indicates the cap domain of endogenous MeFDH1. (**b**) Aligned electron arrays of two MeFDH1 structures. (**c**) Geometrical arrangement of electronically coupled cofactors of the recombinant (left) and endogenous (Right). Cofactors are shown in the stick model. The distances between the cofactors are given in angstroms for edge-to-edge measurements. (**d**) The measurement of the specific activity for formate oxidation. The bar indicates standard deviation. (**e**) The measurement of tungsten content of MeFDH1. The measurement of the endogenous MeFDH1 oxidation activity and metal content is adopted from Eur. J. Biochem. 270(2), 325-333

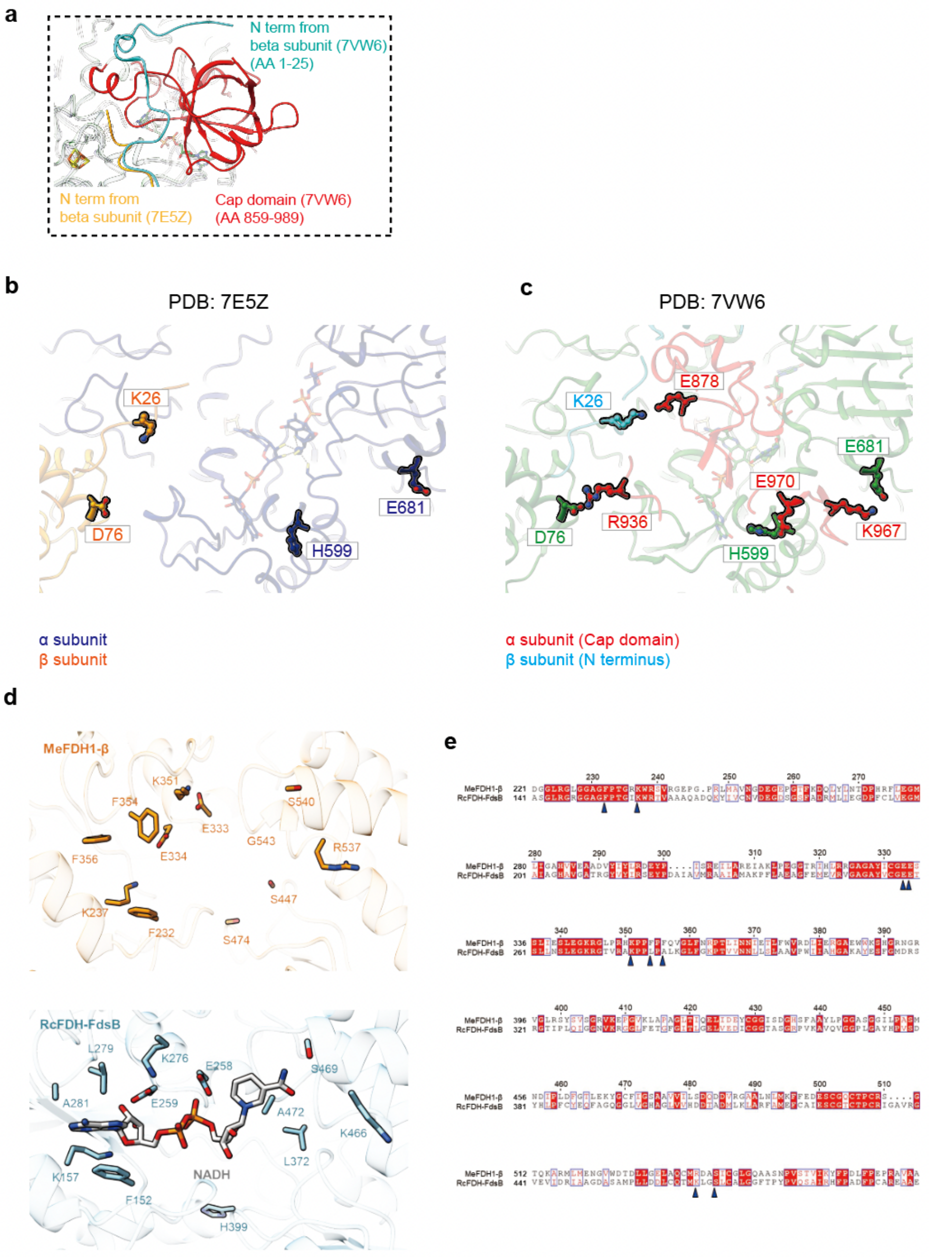

Supplementary Figure S5. The cap domain and NADH binding site of MeFDH1.

(**a**) A comparison between recombinant MeFDH1 (PDB: 7E5Z) and native MeFDH1 (PDB: 7VW6). The N-terminus of the beta subunit (orange:7E5Z, cyan: 7VW6) and the cap domain (PDB:7VW6) are shown. (**b-c**) Residues that interact with the cap domain are shown as ball and stick models. The alpha subunit and beta subunit are colored slate gray and orange for 7E5Z. For 7VW6, the cap domain is colored red, the N-terminus is colored cyan, and the rest domains are colored green. (**d**) Residues involved in NADH binding are drawn as sticks for MeFDH and RcFDH. Sequence alignment of the MeFDH1-β subunit vs. FdsB of RcFDH (UniProtKB/Swiss-Prot accession number D5AQH0). (**e**) The residues interacting with NADH are indicated by arrows.
